# A revision of the genus *Mecistostethus* Marseul (Histeridae, Histerinae, Exosternini)

**DOI:** 10.3897/zookeys.213.3552

**Published:** 2012-08-01

**Authors:** Michael S. Caterino, Alexey K. Tishechkin, Nicolas Dégallier

**Affiliations:** 1 Department of Invertebrate Zoology, Santa Barbara Museum of Natural History, 2559 Puesta del Sol, Santa Barbara, CA 93105 USA; 2 120 rue de Charonne, 75011 Paris France

**Keywords:** Histeridae, Exosternini, *Mecistostethus*, myrmecophily, Neotropical Region

## Abstract

We revise the genus *Mecistostethus* Marseul, sinking the monotypic genus *Tarsilister* Bruch as a junior synonym. *Mecistostethus* contains six valid species: *Mecistostethus pilifer* Marseul, *Mecistostethus loretoensis* (Bruch), **comb. n.**, *Mecistostethus seagorum* **sp. n.**, *Mecistostethus carltoni* **sp. n.**, *Mecistostethus marseuli* **sp. n.**, and *Mecistostethus flechtmanni* **sp. n.** The few existing records show the genus to be widespread in tropical and subtropical South America, from northern Argentina to western Amazonian Ecuador and French Guiana. Only a single host record associates one species with the ant *Pachycondyla striata* Smith (Formicidae: Ponerinae), but it is possible that related ants host all the species.

## Introduction

The genus *Mecistostethus* Marseul is one of the most extremely modified genera of Exosternini in the Neotropics. In fact, like another recently revised genus, *Kaszabister* Mazur (Dégallier et al. 2012), *Mecistostethus* has spent much of its taxonomic history placed in the subfamily Haeteriinae, a group principally composed of highly specialized myrmecophilous and termitophilous inquilines (Mazur 1984, 1997, Helava et al. 1985). Its relationship with Exosternini has been recognized only recently (Mazur 2011), and still remains to be formally supported. *Mecistostethus* was described for a single species, *Mecistostethus pilifer* Marseul from the ‘Amazon’ region, and it has remained monotypic since description. However, a close relative, *Tarsilister loretoensis* Bruch, another monotypic genus described in Haeteriinae, has remained unassociated (though their relationship was suggested by Helava et al. 1985). Here we formally synonymize these two genera, and present descriptions of several new species.

**Figure 1.**
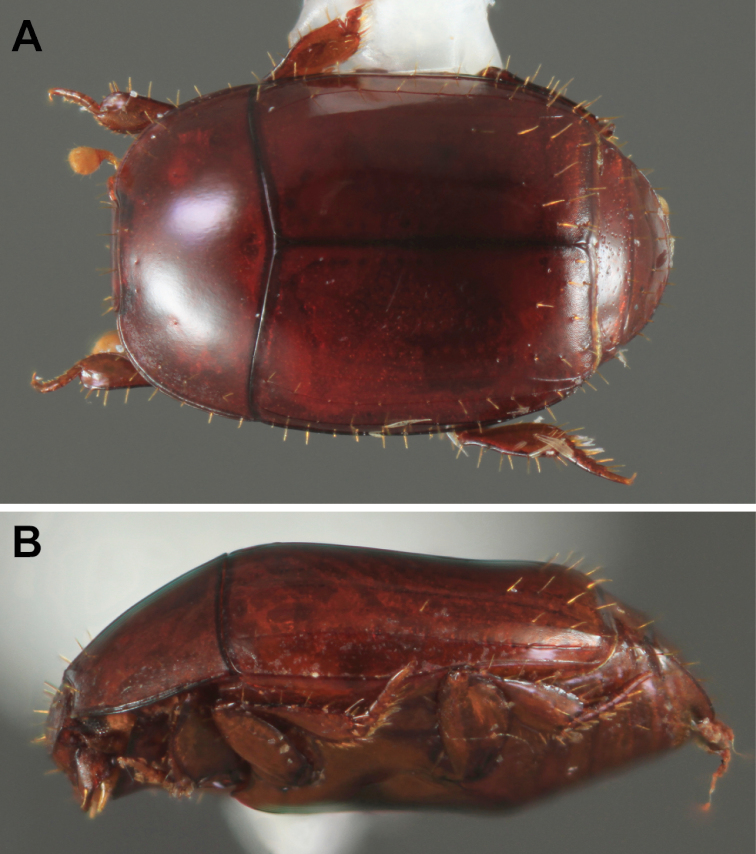
Habitus photographs of *Mecistostethus carltoni* sp. n.A. Dorsal view B. Lateral view.

**Figure 2.**
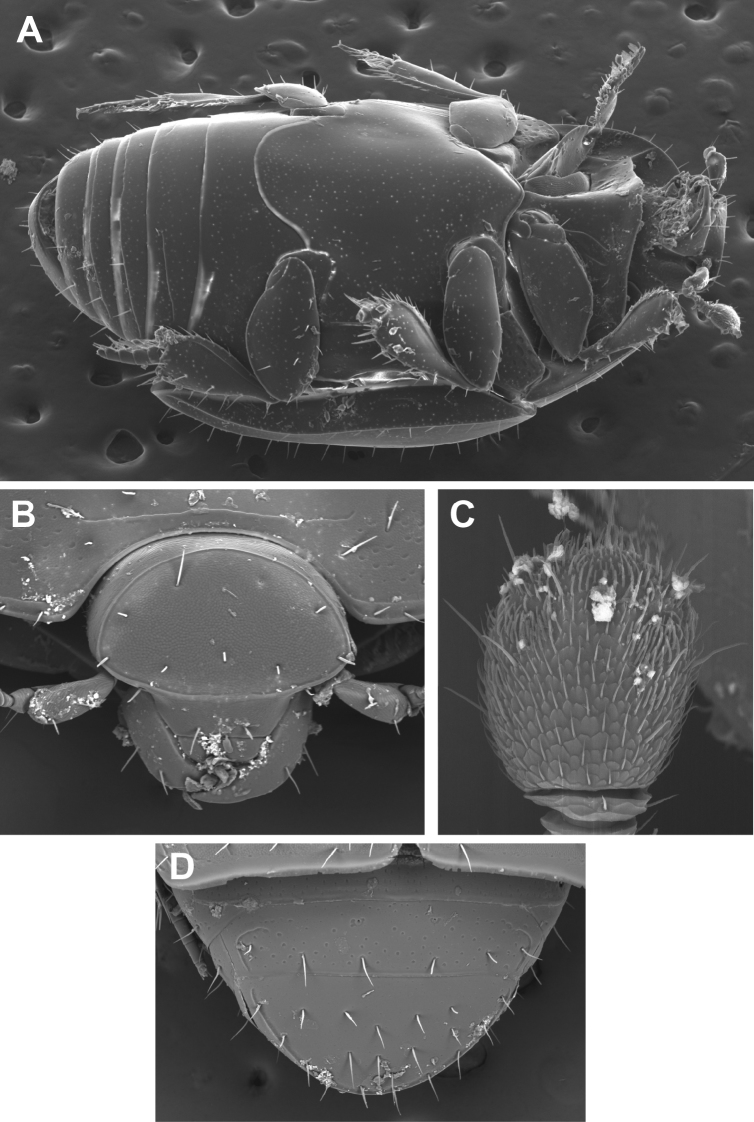
Scanning electron micrographs of *Mecistostethus flechtmanni* sp. n. illustrating generic characters. **A** Ventrolateral view **B** Front of head **C** Antennal club **D** Propygidium and pygidium.

The morphology of *Mecistostethus* (Figs 1, 2) presents some extremely autapomorphic features. Principal among these is the very elongated mesometaventrite (from which the genus name, meaning ‘very long chest’, is derived). In the front the mesoventrite is inflated and projects ventrad and anterad, concealing the base of the prosternal keel. Posteriorly the metaventral margin is broadly arcuate, projecting deeply into the first abdominal ventrite (Fig. 2A). Dorsally, the elytra show a strong medial depression across the posterior half. The body is generally broad, depressed and setose (Fig. 1A). All of these features are very atypical, even for the entire family, and have hindered understanding of relationships. Helava et al. (1985), in their revision of the genera of Haeteriinae, examined only *Tarsilister* and retained it within the subfamily, although they did resolve it as sister of all other haeteriine genera, citing several characters not shared with Haeteriinae as plesiomorphies (narrow antennal scape, tomentose antennal club, presence of tibial spurs, presence of fully articulated coxites with free styli in the females). Only very recent work comprehensively documenting morphological and molecular diversities of both the Exosternini and Haeteriinae (Dégallier et al. 2011; Caterino, Tishechkin, Dégallier, Gomy, and Mazur, in prep.) has produced sufficient character data to conclusively remove this taxon from Haeteriinae and place it unambiguously into Exosternini.

The habits of *Mecistostethus* are largely unknown. While the unusual morphology strongly suggests an inquilinous lifestyle, only a single host record supports this. This record comes from Bruch’s (1932) description of *Tarsilister* (now *Mecistostethus*) *loretoensis*, in which he reports the collection of the unique type in the larval chamber of a nest of *Pachycondyla striata* Smith (Hymenoptera: Formicidae: Ponerinae). The small number of additional specimens available for study have been collected by flight interception traps. Species of Ponerinae are relatively uncommon hosts of histerids in the neotropics, so it might be premature to assume they are the hosts of the other known species. But it is possible, and certainly merits further investigation.

## Materials and methods

The morphological terminology used is that defined by Wenzel and Dybas (1941), supplemented by Helava et al. (1985), Ôhara (1994) and Lawrence et al. (2011). Following histerid conventions, total body length is measured from the anterior margin of the pronotum to the posterior margin of the elytra (to exclude preservation variability in head and pygidial extension), while width is taken at the widest point, generally near the elytral humeri. Conventional imaging was done using a Visionary Digital’s ‘Passport’ portable imaging system, which incorporates a Canon 7D with MP-E 65mm 1–5× macro zoom lens. Images were stacked using Helicon Focus software (www.heliconsoft.com ). SEM imaging was done on a Zeiss EVO 40 scope, and the specimen was sputter coated with gold. Photographs of all type specimens are available through the Encyclopedia of Life (www.eol.org ).

Specimens from the following collections were studied:

CHND The Nicolas Dégallier collection, Paris, France

FMNH The Field Museum, Chicago, USA

MACN Museo Argentino de Ciencias Naturales Bernardino Rivadavia, Buenos Aires, Argentina

MEFEIS Museu de Entomologia, Faculdade de Engenharia, Universidade Estadual Paulista, Ilha Solteira, Brazil

MNHN Museum National d’Histoire Naturelle, Paris, France

## Taxonomy

### Genus Mecistostethus

Marseul, 1870

http://species-id.net/wiki/Mecistostethus

Mecistostethus Marseul 1870: 123.Type species *Mecistostethus pilifer*Marseul 1870: 123, by monotypy.
Tarsilister Bruch 1932: 278. Type species *Tarsilister loretoensis*Bruch 1932: 279, by original designation; NEW SYNONYMY.

#### Diagnosis.

The genus *Mecistostethus* is easily recognized on the basis of numerous autapomorphies, most significantly the elevation of the mesoventrite as a strongly protruding keel (Fig. 2A) (as opposed to the typically coplanar meso+metaventrite and prosternum), as well as the posteriorly arcuate margin of the metaventrite, projecting deeply into the 1^st^ abdominal ventrite (Fig. 2A). In addition the setose body (Fig 1A–B), broadened tibiae (Fig. 2a), convex frons (Fig. 2B), and elytra which are depressed in the posterior third (Fig. 1B), completely lacking dorsal striae 3-5 and sutural stria combine to make this one of the most easily recognizeable New World Histerinae genera.

#### Description.

***Size range***: Length 1.8–2.7mm; width 1.4–2.2mm; ***Body shape*:** Body elongate oval, moderately flattened, rufescent to rufo-piceous, variably microsculptured. ***Head***: Frons strongly convex, with epistoma slightly declivous, disk setose, with or without granulate microsculpture; frontal stria outwardly arcuate and subcarinate when present, absent from some species; subraorbital stria present and continuous with sides of frontal stria; labrum about twice as wide as long, apical margin weakly emarginate; mandibles rather short, lacking subapical teeth; antennal scape elongate, slightly swollen subapically; antennal club oval, tomentose, lacking sutures or distinct annuli, with two small dorsal sensoria near apex of upper surface (Fig. 2C); submentum with sutures weakly impressed, bearing a few setae; mentum flat, nearly twice as broad as long, slightly tapered toward apex, apical margin shallowly emarginate; palpi elongate, with apical palpomeres acuminate. ***Pronotum***: Pronotum with sides rounded, narrowed to apex, anterior emargination simple, prescutellar impression absent; pronotal discal gland openings small, annulate, situated about one-third from anterior margin (just beyond ends of recurved anterior submarginal stria, when present), approximately head-width apart; disk generally with punctures near sides and bearing setae variously arranged; marginal stria complete, free anteriorly, bearing 8-11 setae; lateral submarginal stria forming a shallow depression close to marginal stria; anterior portion of marginal stria continuous with lateral submarginal stria; anterior submarginal stria sometimes present, with ends free and recurved posterolaterally. ***Elytra***: Epipleuron lacking striae; dorsal elytral striae subcarinate and bearing setae; outer subhumeral stria complete, cariniform, forming a lateral elytral margin; inner subhumeral, 1^st^ and 2^nd^ dorsal striae more or less complete and convergent to posterolateral corner; other elytral striae absent. ***Prosternum***: Prosternal lobe short, extending to hypomeron, with medial fragments of marginal stria in some; prosternal keel posteriorly emarginate, but covered by strongly produced mesoventral process; striae of prosternal keel present or absent. ***Mesoventrite***: Mesoventrite strongly elevated (Fig. 2A), subacute anteriorly, projecting over base of prosternum; marginal mesoventral stria complete; mesometaventral stria present or absent. ***Metaventrite***: Posterior margin of metaventrite strongly produced posterad. ***Abdomen***: Abdominal ventrites smooth to faintly punctate; abdominal ventrites 2-5 with stria along posterior margin; propygidium short, flat, with two anteromedial gland openings and lateral marginal striae; pygidium rounded apically, setose, with fine marginal stria (Fig. 2D). ***Legs***: Protrochanter with single seta; protibial margin even, bearing fine marginal spines; protibial spurs present, weak; protarsal setae expanded; male protarsal claws simple; meso- and metatibiae expanded, with even, weakly spinose margins; meso- and metatarsi with numerous ventral setae. ***Male*** (Fig. 5): Paired accessory sclerites present, weak and small; 8^th^ tergite with broad basal and narrower apical emarginations, line of basal membrane attachment complete, just distad basal emargination, ventral apodemes widely separated along midline; 8^th^ sternite with halves separated, apical guides moderately to strongly developed, narrowed apically; 9^th^ tergite with strong ventrolateral apodemes, about one-third from apex; spiculum gastrale (S9) rather narrow, only slightly expanded at base, more weakly sclerotized along midline, with deep, narrow apical emargination, apical flanges not strongly developed; 10^th^ tergite entire, not divided along midline; basal piece slightly elongate, from one-fourth to one-third tegmen length; tegmen narrow, variably expanded to apex, with basolateral carinae converging to delimit a ventral concavity, in some with a thin median keel within this concavity; median lobe from one-fourth to one-half tegmen length. ***Female***: 8^th^ tergite united, emarginate apically, with secondary apicolateral emarginations; 8^th^ sternite divided into one central and two lateral plates, the basal baculi separate, articulated with the lateral plates; 9^th^ sternite present as a median plate, with a sclerotized basal connection to sternite 8; 10^th^ tergite present, undivided; valviferae enlarged at base, articulated with coxites; coxites about one-half length of valvifers, about twice as long as maximum (basal) width, with strong inner apical tooth and much weaker outer one; gonostylus present, setose; bursa copulatrix lumenous, without sclerites; spermatheca and associated glands not examined.

#### Distribution.

The species are exclusively South American, but with scattered records from a surprising variety of biotopes, including Atlantic forests of Santa Catarina, Brazil, cerrado of Mato Grosso do Sul, lowland Amazonian forest of Ecuador and low to mid-elevations on the Guianan shield of French Guiana.

#### Remarks.

All recent collections have been through the use of flight interception traps, and consist solely of male specimens. Only two female specimens of the genus are known, both of *Mecistostethus loretoensis* (Bruch).

##### Key to species

**Table:** 

1	Pronotum with detached anterior submarginal stria in addition to marginal stria (Figs 3A–C, 3F)	2
–	Anterior region of pronotum lacking anterior submarginal stria, with marginal stria only (Figs 3D, E)	5
2	Prosternal striae present (Fig. 4A); anterior submarginal pronotal stria strongly recurved (Fig. 3A); body completely lacking microsculpture	*Mecistostethus loretoensis* (Bruch)
–	Prosternal striae absent (Fig. 4B–D); anterior submarginal pronotal stria variable; microsculpture present at least on frons (usually on pronotum and parts of elytra as well)	3
3	Elytra with stria along apical margin; body larger, >2.5mm, piceous	*Mecistostethus seagorum* sp. n.
–	Elytra lacking stria along apical margin; body smaller, <2.2mm, rufescent	4
4	Elytron with 2^nd^ dorsal stria reaching basal margin; anterior submarginal stria of pronotum strong, with ends curved posterolaterally (Fig. 3F); lateral portion of pronotal disk with conspicuous punctures near edge	*Mecistostethus flechtmanni* sp. n.
–	Elytron with 2^nd^ dorsal stria abbreviated just short of basal margin; anterior submarginal stria of pronotum weak, mostly transverse (Fig. 3C); lateral portion of pronotal disk lacking larger punctures	*Mecistostethus pilifer* Marseul
5	Lateral pronotal discal setae in single submarginal row (Fig. 3E)	*Mecistostethus carltoni* sp. n.
–	Lateral pronotal discal setae not organized in a single row (Fig. 3D)	*Mecistostethus marseuli* sp. n.

### Mecistostethus loretoensis

(Bruch, 1932) comb. n.

http://species-id.net/wiki/Mecistostethus_loretoensis

Figs 3A 4A 7

Tarsilister loretoensis Bruch, 1932: 279.

#### Type material.

**Holotype**, of undetermined sex: «Tarsilister loretoensis Bruch (written by Bruch) C. BRUCH DETERM. (printed)» (label white with black frame) / «Nido de Pachycondyla striata F Sm» (handwritten) / «Typus» (handwritten) / «Est. Exp. Loreto (Misiones - Arg.) [27.32°S, 55.53°W] Dr. A.A. Oglobin» (printed) ; reverse: «20.ix.1931» (handwritten), MACN. Other material: 1 male, locality as for type, 15.VIII.1932, with *Pachycondyla striata*, MACN; 1 female: **BRAZIL: Sta. Catarina:** Nova Teutonia, 27°11'S, 52°23'W, Fritz Plaumann (no date), FMNH-INS0000069073.

#### Diagnosis.

**Figure 3.**
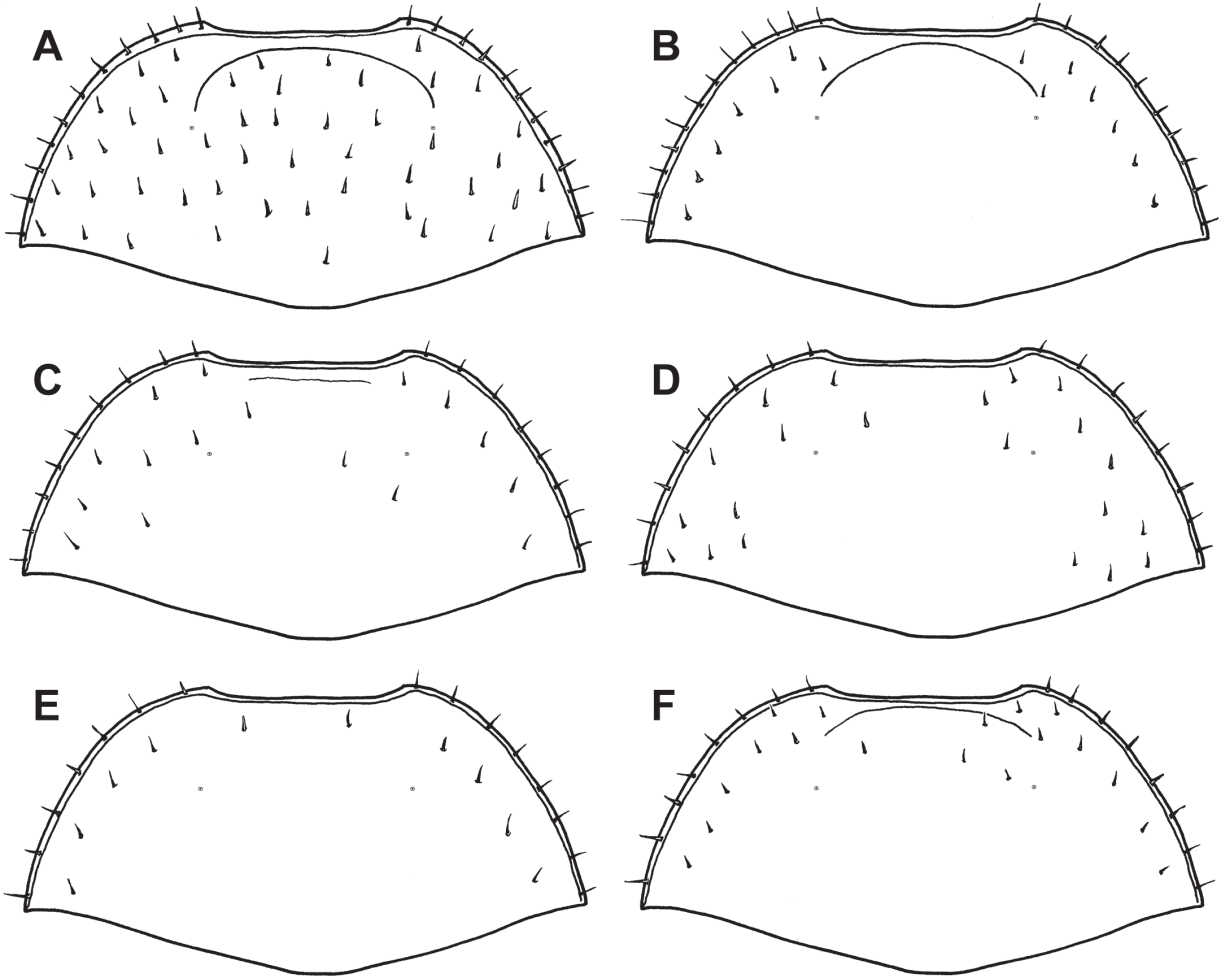
Pronota of all *Mecistostethus* spp. **A** *Mecistostethus loretoensis* **B** *Mecistostethus seagorum* **C** *Mecistostethus pilifer* **D** *Mecistostethus marseuli* **E** *Mecistostethus carltoni* **F** *Mecistostethus flechtmanni*.

**Figure 4.**
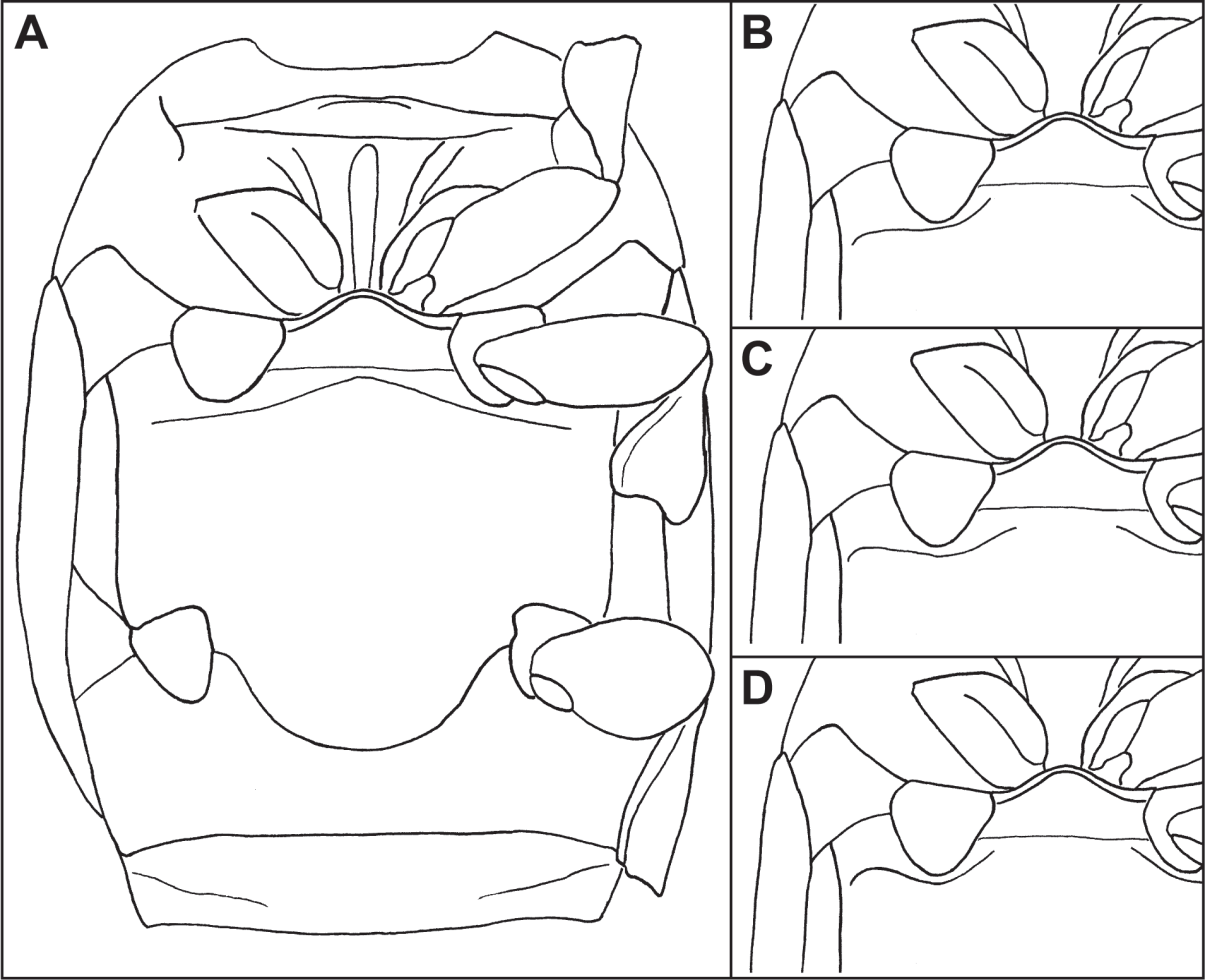
Ventral views of *Mecistostethus* spp. showing striae. **A** Prosternum, meso- and metaventrites of *Mecistostethus loretoensis* **B** Meso- and metaventrites of *Mecistostethus pilifer* (identical in *Mecistostethus marseuli* & *Mecistostethus carltoni*) **C** Meso- and metaventrites of *Mecistostethus seagorum* **D** Meso- and metaventrites of *Mecistostethus flechtmanni*.

Length 2.4-2.6mm, width 2.0–2.1mm (n=3); body completely lacking microsculpture; frontal stria/carina complete; frons and epistoma without microsculpture; anterior submarginal pronotal stria long, ends strongly recurved (Fig. 3A); pronotum with >10 discal setae, scattered on disk without well-defined submarginal row (Fig. 3A); lateral pronotal punctures numerous and conspicuous; prosternal striae present (Fig. 4A); metaventral stria complete at middle (Fig. 4A); elytral stria 1 not continuous, effaced at middle (despite presence of setae); elytral stria 2 barely abbreviated at base; male not examined.

**Figure 5.**
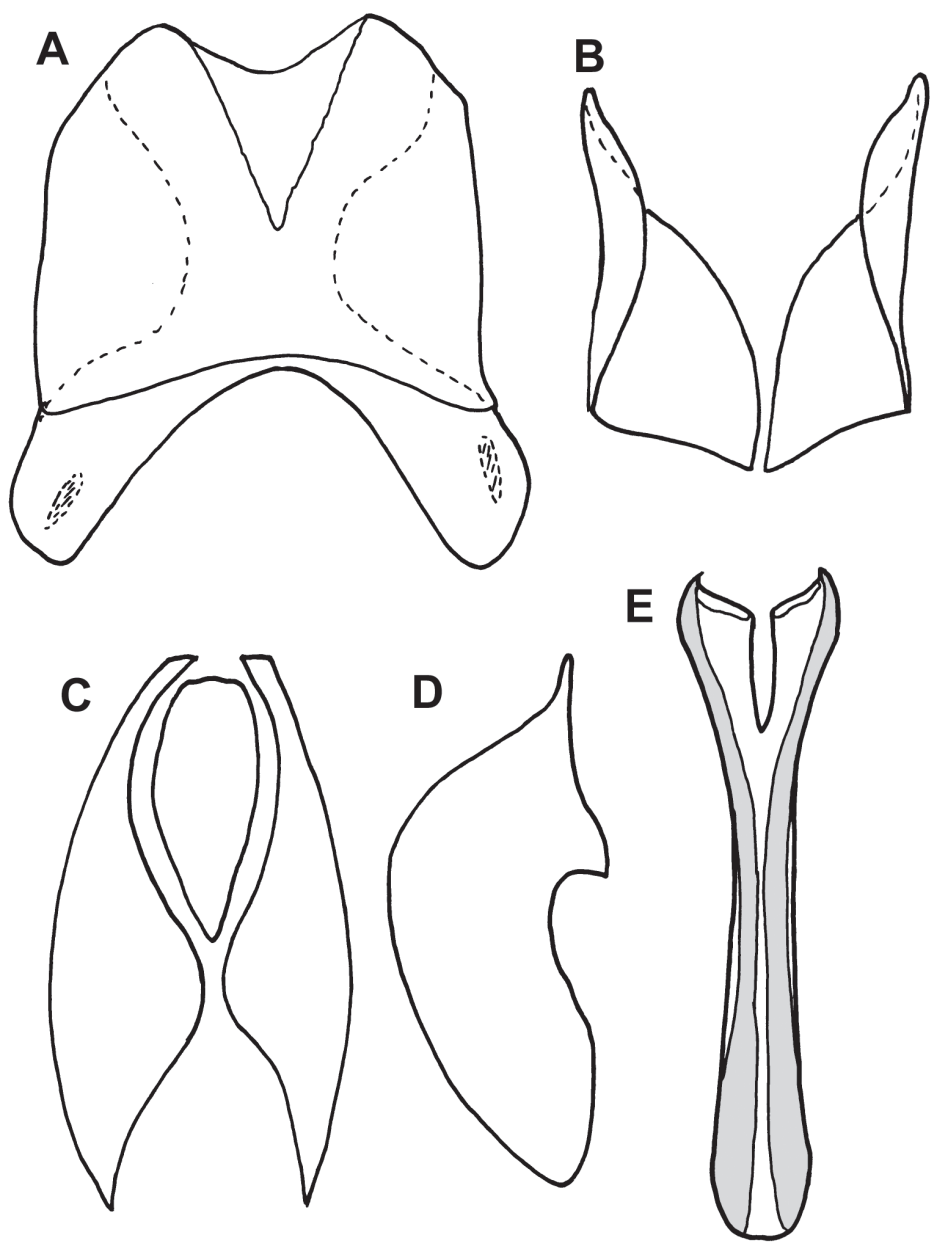
Genital segments 8–10 of *Mecistostethus* (*Mecistostethus carltoni* pictured, others essentially identical). **A** Eighth tergite, dorsal view **B** Eighth sternite, dorsal view **C** Ninth and tenth tergites, dorsal view **D**  Ninth tergite, lateral view **E** Spiculum gastrale (ninth sternite), dorsal view.

#### Distribution.

Known from Atlantic forest areas of Santa Catarina, Brazil, and subtropical forests near the Rio Paraná in Misiones province, Argentina.

#### Remarks.

The type collection from the nest of *Pachycondyla striata* (Ponerinae) provides the only known host record for this genus.

### Mecistostethus seagorum sp. n.

urn:lsid:zoobank.org:act:8EA5060B-4C94-41F9-B46B-7753E0617623

http://species-id.net/wiki/Mecistostethus_seagorum

Figs 3B 4C 6A–B 7

#### Type material.

**Holotype** male: “**GUYANE FR:** Bélvédère de Saül, point de vue, 3°1'22"N, 53°12'34"W, piège vitre, 31 Nov 2010, SEAG” / “Caterino/Tishechkin Exosternini Voucher EXO-01295” / “HOLOTYPE *Mecistostethus seagorum* Caterino, Tishechkin & Dégallier”; deposited in MNHN. **Paratype** male, same locality as type, collected 2.ix.2010; deposited in CHND.

#### Diagnostic description.

**Figure 6.**
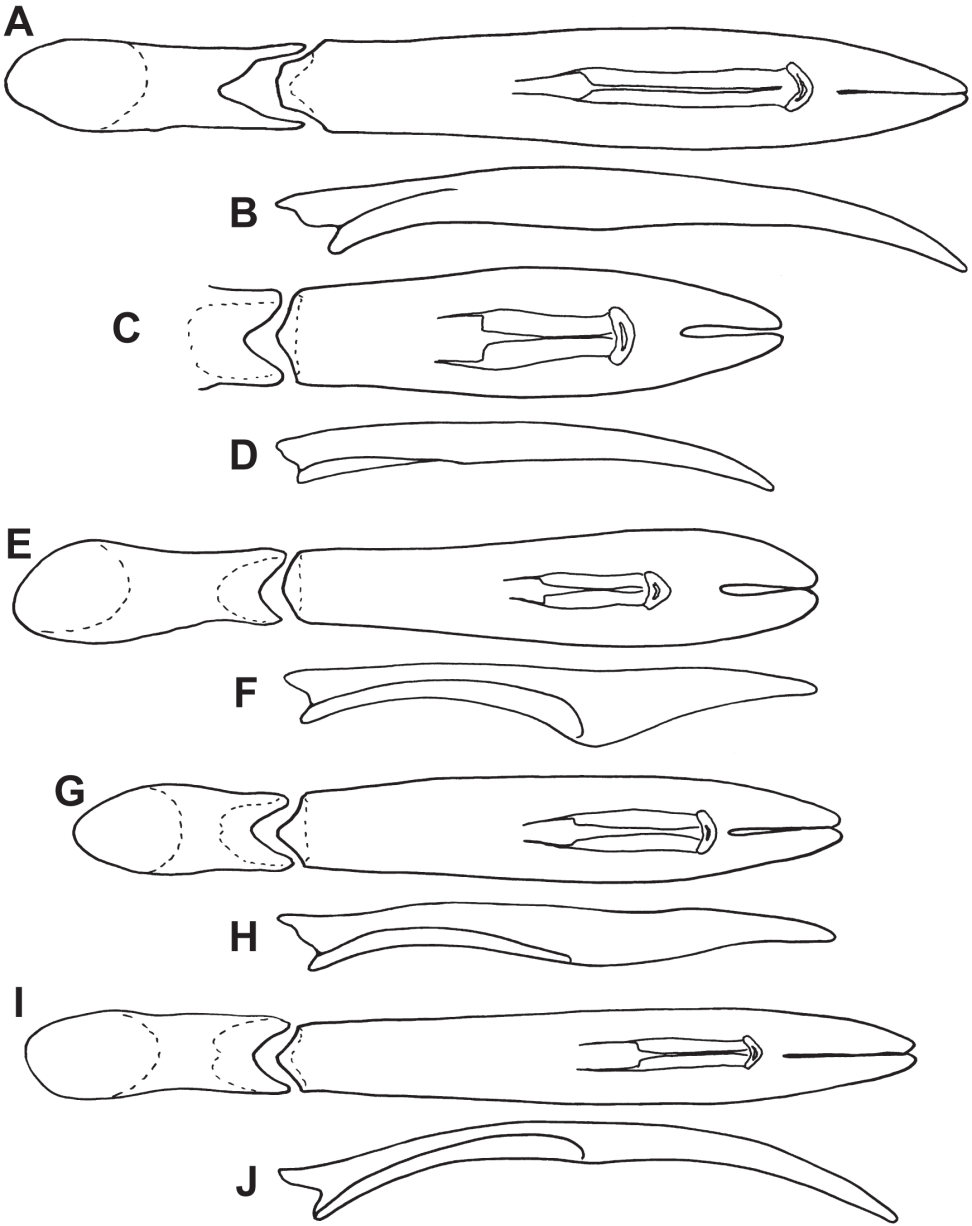
Aedeagi. **A, B** *Mecistostethus seagorum*, dorsal and lateral views, respectively **C, D** *Mecistostethus pilifer*, dorsal and lateral views (basal piece broken basally in type) **E, F** *Mecistostethus marseuli*, dorsal and lateral views **G, H** *Mecistostethus carltoni*, dorsal and lateral views **I, J** *Mecistostethus flechtmanni*, dorsal and lateral views.

Length 2.7mm, width 2.2mm; frontal stria complete; frons and epistoma with microsculpture; anterior pronotal stria long, divergent (Fig. 3B); pronotum with ~10 setae on disk, arranged in a well-defined submarginal row (Fig. 3B); lateral pronotal punctures present, but extremely faint; pronotal micro-sculpture present on entire disk; prosternal striae absent (Fig. 4C); metaventral stria interrupted at middle (Fig. 4C); elytral microsculpture absent; elytral stria 2 complete, with numerous setae; elytral striae 1 and 2 reaching base; elytral stria 2 united with an apical marginal stria reaching nearly to suture; tegmen (Figs 6A–B) narrowed to base and apex, widest about one-fourth from apex, in lateral view nearly evenly curved to apex, with weak ventral swelling just basad midpoint, basoventral concavity occupying less than basal third, poorly defined, lateral carinae rapidly weakened from base; median lobe relatively long, nearly one-half tegmen length.

**Figure 7.**
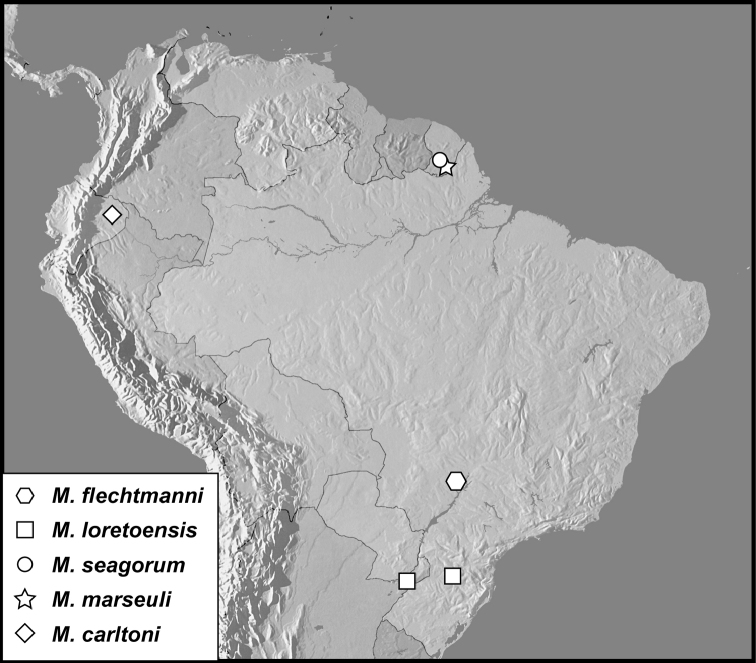
Map showing all collecting localities. The exact type locality for *Mecistostethus pilifer*, ‘Amazones’, is too imprecise to be mapped.

#### Distribution.

This species is only known from the type locality, in south-central French Guiana.

#### Etymology.

This species name recognizes the impressive efforts of the Société Entomologique Antilles Guyane (SEAG) to inventory the rich insect biodiversity of the Guianas (http://insectafgseag.myspecies.info/ )

### Mecistostethus pilifer

Marseul, 1870

http://species-id.net/wiki/Mecistostethus_pilifer

Figs 3C 4B 6C–D 7

Mecistostethus pilifer Marseul, 1870: 123

#### Type material.

Lectotype male designated herein in order to fix the status of the name-bearing specimen: (barely legible green disk) “N[?], Mecistost. pilifer, Amazones, Bates, ’69[5?]” / “TYPE” / “MUSEUM PARIS, COLL DE MARSEUL 1890” / “LECTOTYPE” / “Mecistostethus pilifer Marseul, 1870 Lectotype N. DÉGALLIER” / “LECTOTYPE Mecistostethus pilifer Marseul, M.S. Caterino & A.K. Tishechkin des. 2010”; MNHN.

#### Diagnosis.

Small, length 1.9mm, width 1.5mm;frontal stria/carina complete**;** frons and epistoma with microsculpture; anterior pronotal stria short, weak, barely divergent from margin (Fig. 3C); pronotum with >10 discal setae (despite many evidently broken off of type), with several scattered setae in addition to well-defined submarginal row (Fig. 3C); pronotal microsculpture gradually more conspicuous to front and sides, inconspicuous at base**;** lateral pronotal punctures absent (aside from setigerous punctures); prosternal striae absent (Fig. 4B); metaventral stria interrupted at middle (Fig. 4B); elytral microsculpture extremely faint, visible only near apex; elytra with stria 1 complete, bearing numerous setae, stria 2 barely abbreviated at base; aedeagus (Fig. 6C) relatively short, with sides rounded, almost evenly tapering basally and apically; tegmen quite flat, with apex only very weakly curved ventrad (Fig. 6D); basoventral concavity shallow but well defined, with basolateral carinae and fine ventral keel present; median lobe slightly over half tegmen length.

#### Distribution.

This species is known only from the vague type locality: “Amazones”.

### Mecistostethus marseuli sp. n.

urn:lsid:zoobank.org:act:C73F8B05-10C0-4AEB-8057-63792297A5C5

http://species-id.net/wiki/Mecistostethus_marseuli

Figs 3D 4B 6E–F 7

#### Type material.

**Holotype** male: “**GUYANE FR:** Itoupé, Mont tabulaire alt. 600m, 3°1.38'N, 53°5.73'W, piège d’interception 3, 23 Mar 2010 SEAG leg.” / “Caterino/Tishechkin Exosternini Voucher EXO-00498” / “HOLOTYPE *Mecistostethus marseuli* Caterino, Tishechkin & Dégallier”; deposited in MNHN.

#### Diagnostic description.

Length 2.0mm, width 1.5mm; frontal stria largely effaced, not prominently carinate; frons and epistoma with microsculpture; anterior submarginal pronotal stria absent; pronotum with >10 discal setae, scattered on disk in addition to submarginal row (Fig. 3D); lateral pronotal punctures absent; pronotal microsculpture more conspicuous at sides, lacking at base; prosternal striae absent; metaventral stria interrupted at middle (Fig. 4B); microsculpture at apex of elytra conspicuous; elytral stria 1 complete, with numerous setae; elytral striae 1 and 2 abbreviated at base; tegmen (Figs 6E–F) widest near apex, tapering in basal two-thirds, basoventral concavity well developed, occupying basal three-fifths, delimited by strong basolateral carinae and with conspicuous fine median keel; tegmen with strong ventral swelling just apicad midpoint; median lobe only about one-fourth tegmen length.

#### Distribution.

This species is only known from the type locality, in southeastern French Guiana.

#### Etymology.

This species is named for French entomologist Sylvain Auguste de Marseul (1812–1890), whose superb work on the family Histeridae remains almost unequaled to this day.

### Mecistostethus carltoni sp. n.

urn:lsid:zoobank.org:act:6E00C1D7-4A32-4555-B6D5-C3A81F5D9B1D

http://species-id.net/wiki/Mecistostethus_carltoni

Figs 1 3E 4B 6G–H 7

#### Type material.

**Holotype** male: “**ECUADOR**: Napo, Yasuní Res.Stn. on mid. Rio Tiputini, 0°40.5'S, 76°24'W. F.I.T.#M1, 7-13 Jul 1999. AKT # 080, C.Carlton & A.Tishechkin” / “LSAM 0012929” / “HOLOTYPE *Mecistostethus carltoni* Caterino, Tishechkin & Dégallier”; deposited in FMNH.

#### Diagnostic description.

Length 1.8mm, width 1.4mm; frontal stria interrupted, effaced at middle, not prominently carinate; frons and epistoma with microsculpture; anterior submarginal pronotal stria absent (Fig. 3E); pronotum with <10 discal setae, arranged in submarginal row only (Fig. 3E); lateral pronotal punctures present, but extremely faint; lateral pronotal microsculpture discrete; prosternal striae absent (Fig. 4B); metaventral stria interrupted at middle (Fig. 4B); microsculpture at apex of elytra conspicuous; elytral stria 1 complete, with numerous setae; elytral striae 1 and 2 reaching elytral base; tegmen (Figs 6G–H) widest just distad midpoint, weakly tapering in basal half, basoventral concavity moderately well developed, occupying basal half, with fine median keel; tegmen with moderate ventral swelling near midpoint, only weakly curved toward apex; median lobe only about one-fourth tegmen length.

#### Distribution.

This species is known only from the type locality, in lowland Amazonian rainforest in eastern Ecuador.

#### Remarks.

This species is named in honor of Dr. Chris Carlton, director of the Louisiana State Arthropod Museum (LSAM), leader of the field trip on which the type of this species was caught, and AKT’s doctoral advisor.

### Mecistostethus flechtmanni sp. n.

urn:lsid:zoobank.org:act:3D9C9A02-30E1-4634-9458-ECDB14FF3EC0

http://species-id.net/wiki/Mecistostethus_flechtmanni

Figs 2 3F 4D 6I–J 7

#### Type material.

**Holotype** male: “**BRAZIL: Mato Grosso do Sul**, cerradão fragment nr. Selviria at 20°20'10"S, 51°24'36"W, Window trap, ground level, trail 1, 17.xii.2010, C.A.H. Flechtmann” / “Caterino/Tishechkin Exosternini Voucher EXO-00644” / “HOLOTYPE *Mecistostethus flechtmanni* Caterino, Tishechkin & Dégallier”, in MEFEIS. **Paratypes** (3 males): same locality as type, collected 14.i.2011 and 28.i.2011 by CAH Flechtmann, and 31.xi-3.xii.2011 by MSC and AKT (DNA Voucher EXO-00933, extract MSC-2274), in MEFEIS, FMNH.

#### Diagnostic description.

Length 1.8-2.0mm, width 1.4-1.6mm; frontal stria complete but weak at middle (Fig. 2B); frons and epistoma with microsculpture; anterior submarginal pronotal stria long, recurved posterad at apices (Fig. 3F); lateral pronotal punctures present; pronotum with <10 discal setae present on disk in addition to submarginal row (Fig. 3F); prosternal striae absent (Fig. 4D); metaventral stria interrupted at middle (Fig. 4D); microsculpture at apex of elytra conspicuous; elytral stria 1 complete, with few setae, mainly near apex; elytral stria 1 and 2 reaching elytral base; tegmen (Figs 6I–J) narrow, weakly tapered in basal half, constant in width in most of apical half, with shallow but strongly delimited basoventral concavity in just less than basal one-half; with very weak ventral swelling near midpoint; median lobe short, about one-fourth tegmen length.

#### Distribution.

This species is known only from the type locality, collected in a fragment of «cerradão» forest close to the Paraná River in extreme eastern Mato Grosso do Sul. This forest is a relatively moist, taller and denser form of the cerrado subtropical biome.

#### Remarks.

This species is named for the collector of most of the type series, and our gracious host during a productive visit to the site, Dr. Carlos Flechtmann of Universidade Estadual Paulista (Department of Plant Protection, FEIS/UNESP, Ilha Solteira campus).

## Discussion

It is remarkable how few specimens of *Mecistostethus* have come to light, just over 10 in the 140 years since it was first discovered. This suggests a highly cryptic and unusual habit. While myrmecophily traditionally fell into this category, recent years of focused collecting by ourselves and colleagues has gradually produced a reasonable wealth of specimens for many formerly rare myrmecophilous histerids. *Mecistostethus*, however, remains among the exceptions. Many of the ‘common’ myrmecophiles have been revealed to have relatively common and abundant hosts, especially the neotropical army ants (Ecitoninae). The apparent association of *Mecistostethus* with *Pachycondyla* spp., which generally have much smaller colonies (da Silva-Melo and Giannotti, 2010), more or less fits with this picture. Their colonies are more difficult to locate, and have received much less attention from collectors. It is hoped that focused future efforts will help fill in the intriguing picture of *Mecistostethus* biology.

## Supplementary Material

XML Treatment for Mecistostethus

XML Treatment for Mecistostethus loretoensis

XML Treatment for Mecistostethus seagorum

XML Treatment for Mecistostethus pilifer

XML Treatment for Mecistostethus marseuli

XML Treatment for Mecistostethus carltoni

XML Treatment for Mecistostethus flechtmanni
